# Molecular Identification of *Leishmania* Species Using Samples Obtained from Negative Stained Smears

**Published:** 2013

**Authors:** MA Mohaghegh, A Fata, GH Salehi, F Berenji, M Mousavi bazzaz, H Rafatpanah, M Parian, A Movahedi

**Affiliations:** 1 Department of Medical Parasitology and Mycology, School of Medicine, Mashhad University of Medical Sciences, Mashhad, Iran; 2 Cutaneous Leishmaniasis Research Center, Emam Reza Hospital, School of Medicine, Mashhad University of Medical Sciences, Mashhad, Iran; 3 Department of Community Medicine, School of Medicine, Mashhad University of Medical Sciences, Mashhad, Iran; 4 Inflammation and Inflammatory Diseases Research Center, Mashhad University of Medical Sciences, Mashhad, Iran

**Keywords:** Cutaneous Leishmaniasis, Direct smear, *Leishmania tropica*, PCR

## Abstract

**Background:**

Cutaneous Leishmaniasis (CL) is a parasitic skin disease. Diagnosis primarily is based on clinical signs and microscopic observation of parasite on direct stained smears or tissue sections. Sensitivity of direct smear is not as high as molecular methods. The aim of this study was to identify and characterize *Leishmania* species among the negative direct smears obtained from skin ulcers suspected to CL by PCR method.

**Methods:**

Among 81 patients with suspicious skin lesions to CL referred to the Parasitology lab, negative Giemsa stained smears were collected. DNA extraction performed by scraping stained smears, then PCR was performed.

**Results:**

Among the DNA extracted from smears, *L. tropica* was isolated from 9 (11.1%) of the smears and *L.major* was not isolated from any samples.

**Conclusion:**

Direct microscopy on stained smears for diagnosis of leishmaniasis is not enough accurate. PCR is recommended for clinically suspected lesions with negative result of direct smear.

## Introduction

Cutaneous Leishmaniasis (CL) is a parasitic skin disease, endemic worldwide (1). To select appropriate treatment, definite and early diagnosis of the parasite is important (2–4). Diagnosis primarily is based on clinical signs and microscopic observation of parasite on direct stained smears or tissue sections (4). Direct microscopic examination and culture are routine diagnostic routes in endemic regions (3, 5). Improper sampling, staining and using old and low resolution microscopes interfere with accurate diagnosis. On the other hand, the sensitivity of direct smear is not as high as molecular methods. In many smears the parasite may be present but not easy to be observed.

An excellent target for a sensitive and rapid detection method is the kinetoplast mini-circle DNA (KDNA), which can be detected by PCR method. The KDNA have been used as targets for selective amplification of parasite DNA in various studies (6–8).

To perform PCR method many investigators have used samples obtained from positive direct spears, but rare reports about performing PCR on samples obtained from negative direct smears are available (9, 10).

To identify *Leishmania* species among the negative direct smears obtained from skin ulcers suspected to CL this study was undertaken over a period of 9 months in The Department of Parasitology, Emam Reza Hospital in Mashhad, Iran.

## Materials and Methods

### Sampling

Study population was 81 persons who had at least one skin ulcer suspected to CL and referred to the Parasitology Department of Emam Reza Hospital, Mashhad, Iran during August 2011 to April 2012. A questionnaire containing demographic and clinical information completed for each individual. Standard direct Giemsa stained smear obtained from skin ulcers of each patient. Low and high power direct microscopy performed by laboratory technician and controlled by medical parasitologist. Negative direct smears stored in a clean box for PCR examination.

### DNA extraction and PCR analysis

DNA extraction was performed using scraped samples on microscope slide plus standard extraction kit (Ge Net Bio, Korea) according to manufacturer's instructions. A smear prepared by a drop of distilled water was also used as negative control for PCR. The two synthetic oligonucleotide primers of KDNA pattern of *L. donovani* used were with sequences F: (5’ TCGCAGAACGCCCCTACC’ 3) and R: (5’ AGGGGTTGGTGTAAAATAGG 3’) according to that described by Martinez et al. (10). The primers prepared by (Tuba-Negin.Iran) the primers investigated by Blast software. The amplification carried out according to instructions of PCR kit provider (Ge Net Bio, Korea). The test was set up at different concentrations of MgCl2 (0.5 to 4 µM). Finally, the best bands were observed 2µl of MgCl2 for both species. The reaction mixtures were 25µl which at the end 5 µl increased of DNA samples.

Amplification was performed in a thermal cycler (ASTEC-PC818) 38 cycles of denaturation at 94 °C for one minute, annealing at 60 °C for 45 second, extension at 72 °C for one minute and final extension at 72 °C for 7 minutes. Two standard samples of parasites (strain MRHO/IR/75/ER of *L. major* and the strain MHOM/IR/01/yaza of *L. tropica*) and a negative control sample were used to monitor the reaction. PCR products were analyzed by 1.5% agarose gel electrophoresis and observed by UVdoc system (Doc-008.XD). DNA replication pattern of *Leishmania* produced bands for *L. major* / 615bp and for *L.tropica* /744bp with these primers. To confirm the integrity extracted DNA, all negative samples by PCR with the primers, beta globulin human gene in the same condition for *Leishmania* were PCR. All negative samples showed a clear band with this gene. The results were analyzed by SPSS 16.

## Results

Of 81 samples obtained from negative direct smears, examined by PCR, *L. tropica* was isolated from 9 (11.1%) samples. Among 81 persons who had skin ulcers 48 (59.3%) were male and 33 (40.7%) were female. The youngest individual was a one year male infant and the oldest was a 68 years old female. Of them 42 (52%) came from rural areas and 39 (48%) were from urban regions.

Most of the lesions were located on exposed organs such as hands (43%), face (31%) and feet (22%). The clinical presentation of skin lesions highly suspected to CL and microscopic pictures of direct smears studied. (Tables 1, 2)

**Table 1:** Clinical presentation of skin lesions in 81 persons highly suspected to CL according to the result of PCR

Type of lesion	No. (%)	PCR+
Papule	11(13.6)	4
Nodule	34(42.0)	1
Ulcer	33(40.7)	3
Lupoid	3 (3.7)	1
Total	81 (100)	9

**Table 2:** Microscopic picture of negative direct smears obtained from skin lesions with primary clinical diagnosis for Cutaneous Leishmaniasis in relation to the result of PCR

Microscopic picture	No. (%)	PCR+
Granulomatose	42(51.9)	5
Suppurative	22 (27.1)	2
Without cell infiltration	17 (21)	2
Total	81(100)	9

## Discussion

Preparation of direct smear is a simple and reliable method and commonly used in all medical laboratories, but it is not enough sensitive to give an accurate and definite result.

The reason why direct smear cannot be used as a gold standard depends on several facts.

1. The quantity and quality of specimen obtained from the lesion.
2. The technique of preparation and staining to provide a good smear.
3. The quality of microscopic resolution and field.
4. The experience of microscopist.

Bacterial and especially fungal contamination of NNN cultures is one of the most important problems in parasitology laboratories. Decrease in size of *Leishman* body during procedures for preparation of biopsy sections, makes it difficult to recognize the parasite, especially when the number of parasites is few.

In one study, comparison of four laboratory techniques in diagnosis of *Leishman* body on more than 280 samples showed that the accuracy of direct smear is more than culture, Leishmanin skin test (LST) and biopsy in that order (11).

PCR is a highly sensitive molecular method for diagnosis of CL in comparison with direct smears, cultures and immune-serological test (12–15). In this study, DNA extraction was performed by the specimen obtained from scraping of negative direct smears. The reports about molecular identification of *Leishmania* on samples obtained from negative direct smears or negative skin biopsies are very rare. In only one available report, *Leishmania* has been identified in 5 negative direct smears out of 30 (16.7%) (18). In another study which performed on samples obtained from positive smears, the result of PCR was 95% positive (16).

In the present study, we used KDNA of Parasite as a source of DNA. In similar studies the same results obtained (13, 17). *Leishmania* has been identified in 24 out of 29(82.7%) biopsy samples of skin lesions previously reported as negative result for presence of *Leishmania* (19). PCR is more sensitive method than direct smear than biopsy. In the present study, 9 out of 81(11.1%) samples obtained from negative direct smears were positive by PCR which confirm the higher sensitivity of molecular method in comparison with direct stained smear.

## Conclusion

PCR, as a valuable method, is very useful for evaluation of negative direct stained smears. It is also reconfirmed that the dominant species of *Leishmania* in Mashhad is *L. tropica* as reported in previous studies (12, 20–22). We still need to do further studies on negative samples by using PCR method to present a definite conclusion about the validity of different methods for identification of *Leishmania*.
